# High-resolution and Deep Phylogenetic Reconstruction of Ancestral States from Large Transcriptomic Data Sets

**DOI:** 10.21769/BioProtoc.3566

**Published:** 2020-03-20

**Authors:** Sumanth Kumar Mutte, Dolf Weijers

**Affiliations:** Laboratory of Biochemistry, Wageningen University, 6708WE, Wageningen, the Netherlands

**Keywords:** Phylogenomics, OneKP, MMETSP, Plants, Phylogenetics, Evolution, Transcriptome

## Abstract

Phylogenetics is an important area of evolutionary biology that helps to understand the origin and divergence of genes, genomes and species. Building meaningful phylogenetic trees is needed for the accurate reconstruction of the past. To achieve a correct phylogenetic understanding of genes or proteins, reliable and robust methods are needed to construct meaningful trees. With the rapidly increasing availability of genome and transcriptome sequencing data, there is a need for efficient and accurate methodologies for ancestral state reconstruction. Currently available methods are mostly specific for certain gene families, and require substantial adaptation for their application to other gene families. Hence, a generalized framework is essential to utilize large transcriptome resources such as OneKP and MMETSP. Here, we have developed a flexible yet efficient method, based on core strengths such as emphasis on being inclusive in homolog selection, and defining orthologs based on multi-layered inferences. We illustrate how specific steps can be modified to fit the needs of any protein family under consideration. We also demonstrate the success of this protocol by studying and testing the orthologs in various gene families. Taken together, we present a protocol for reconstructing the ancestral states of various domains and proteins across multiple kingdoms of eukaryotes, using thousands of transcriptomes.

## Background

Phylogenetic trees are fundamental to understanding the evolution of genes, gene families, species, phyla and even kingdoms. They help to depict the diversity and also resolve the differences at various levels. For example, at protein level, they help us to identify orthologous groups based on amino acid differences across various species. Earlier, phylogenetic trees were constructed based on few gene/protein sequences from limited numbers of species. With the ever-growing sequencing data, as more and more genomes and transcriptomes are becoming accessible, there is tremendous potential for *e.g.*, discovery of new lineages, ‘gap-filling’ in phylogenies and hence, an improved understanding of biology (Levy and Myers, 2016; Burki *et al.*, 2019).

 In the last decade, many efforts have been made towards defining transcriptomes of hundreds (or even thousands) of species due to the popularity of RNA-Seq (Stark *et al.*, 2019). Transcriptomes provide a quick insight into the (expressed) gene content of a genome. Even though the individual transcriptomes do not cover the entire gene content of an organism, combining them from multiple cells, tissues and conditions, may comprise the majority of the transcribed genes of that species. Hence, it is a relatively straightforward approach to sequence and assemble a transcriptome without *a priori* knowledge of the genome. The current-day long-read and single-cell RNA-sequencing technologies make it even easier to build a complete transcriptome (Wang *et al.*, 2016). Utilizing these technological advances, two large transcriptome sequencing projects, 1000 plant transcriptomes (OneKP; Carpenter *et al.*, 2019; One Thousand Plant Transcriptomes Initiative, 2019) and Marine Micro Eukaryote Transcriptome Sequencing Project (MMETSP; Keeling *et al.*, 2014), were developed. OneKP represents the majority of the land plants and algal groups, whereas MMETSP covers majority of the SAR group and other (unidentified) phyla in Chromista.

 From their inception, diverse approaches have been developed and applied to these transcriptomes and estimate the ancestral states of various genes across multiple classes, families and even phyla (Li *et al.*, 2014; Wickett *et al.*, 2014; Yerramsetty *et al.*, 2016). The majority of these methods focus on one gene family, and need substantial modifications in methodology to apply them to other gene families. Moreover, the methods used are neither inclusive nor robust in terms of multi-layered inferences. The orthologous inferences are based on only one evidence, Best Bi-directional Hit or protein domains or simple phylogenies based on few genomes. To overcome these disadvantages, we developed a unified framework to build high-resolution phylogenies that utilize the rich OneKP and MMETSP transcriptome resources. This new method is not only inclusive, but also utilizes multi-layered orthology to interpret phylogenies with high confidence, leading to the identification of new (sub-)classes of orthologs.

**Overview of the protocol**

The current protocol is developed to reconstruct ancestral states and high-resolution phylogenetic trees of various gene families using transcriptomes and/or proteomes. Ancestral state represents the minimal gene complement at each evolutionary node, where species-specific gene duplications and (or) losses would have modified the gene complement in individual species. Hence, selecting the correct, orthologous as well as diverse, sequences is a crucial step in such a deep phylogenetic tree construction. This protocol is built on three core strengths: (1) Inclusive: Include more sequences at the start with liberal parameters, and remove sequences as one goes through various steps in the pipeline, resulting in a high-quality logical sequence set for phylogenetic tree construction. (2) Multi-layered: Multiple levels of orthology confirmation, *i.e.*, based on the domain architecture, reciprocal BLAST and the phylogenetic tree. (3) Robust: No limitations on length of the protein or the number of sequences used in the phylogeny, with suggestions on alternate analysis packages tested in various steps. Overall, the protocol comprises 14 steps that are divided into three sections: Homolog identification (Steps 1-5), Ortholog detection (Steps 6-8) and Phylogeny construction (Steps 9-14). All the general parameters and recommendations for the respective steps are indicated below.

## Equipment

1. Linux machine
  Computer set-up: Majority of the mentioned programs in Software section run only on Linux environment; hence it is recommended to perform the analysis on a Linux machine with access to the BASH shell (terminal). The average time needed to perform the analysis for a gene family is 1-1.5 days on a generic Linux workstation with 64 GB RAM and 8-core processor setup. The disk space needed for this analysis is less than 1 GB.

## Software

1. tblastn and blastp from BLAST+ module v2.9.0 (Camacho *et al.*, 2009) (ftp://ftp.ncbi.nlm.nih.gov/blast/executables/blast+/LATEST/)
2. faSomeRecords: Linux binary from UCSC (http://hgdownload.cse.ucsc.edu/admin/exe/linux.x86_64/)
3. TransDecoder v5.5.0 (Haas *et al.*, 2013) (transdecoder.github.io)
4. MEME motif discovery v5.1.0 (Bailey *et al.*, 2009) (http://meme-suite.org/)
5. ScanProsite web-tool (https://prosite.expasy.org/scanprosite)
6. InterProScan v5.38-76.0 (Jones *et al.*, 2014) (https://github.com/ebi-pf-team/interproscan)
7. MAFFT v7 (Katoh and Standley, 2013) (https://mafft.cbrc.jp/alignment/software/)
8. JalView (Waterhouse *et al.*, 2009) (https://www.jalview.org/)
9. ModelFinder (Kalyaanamoorthy *et al.*, 2017) (accessed as in-built module from IQ-TREE)
10. ModelTest-NG (Darriba *et al.*, 2020) (https://github.com/ddarriba/modeltest)
11. PartitionFinder v2 (Lanfear *et al.*, 2012) (http://www.robertlanfear.com/partitionfinder/)
12. IQ-TREE v1.6.12 (Nguyen *et al.*, 2015) (http://www.iqtree.org)
13. RAxML v8 (Stamatakis, 2014) (https://cme.h-its.org/exelixis/web/software/raxml/index.html)
14. PhyML v3.3 (Guindon *et al.*, 2010) (https://github.com/stephaneguindon/phyml)
15. MrBayes v3.2.7 (Ronquist *et al.*, 2012) (https://github.com/NBISweden/MrBayes)
16. iTOL v4 (Letunic and Bork, 2019) (https://itol.embl.de)
17. Linux BASH shell (terminal) ‘cut, sort and uniq’ functions (https://tiswww.case.edu/php/chet/bash/bashref.html)
18. Scripts used for automating certain steps in the protocol are available through GitHub (https://github.com/sumanthmutte/Phylogenomics)
  **Data**
  1. OneKP dataset (1000 plant transcriptomes project): Contains 1341 transcriptomes from 1179 species covering all the major classes of land plants, green algae, red algae and glaucophytes (Carpenter *et al.*, 2019; One Thousand Plant Transcriptomes Initiative, 2019); http://datacommons.cyverse.org/browse/iplant/home/shared/commons_repo/curated/oneKP_capstone_2019
  2. MMETSP dataset (Marine Microbial Eukaryote Transcriptome Sequencing Project): Contains 678 transcriptomes from 410 species covering all the major classes of Stramenopila and Alveolata (SAR group) and many unclassified (unicellular) marine eukaryotes (Keeling *et al.*, 2014); https://gold.jgi.doe.gov/study?id=Gs0128947

## Procedure

**Figure 1.**
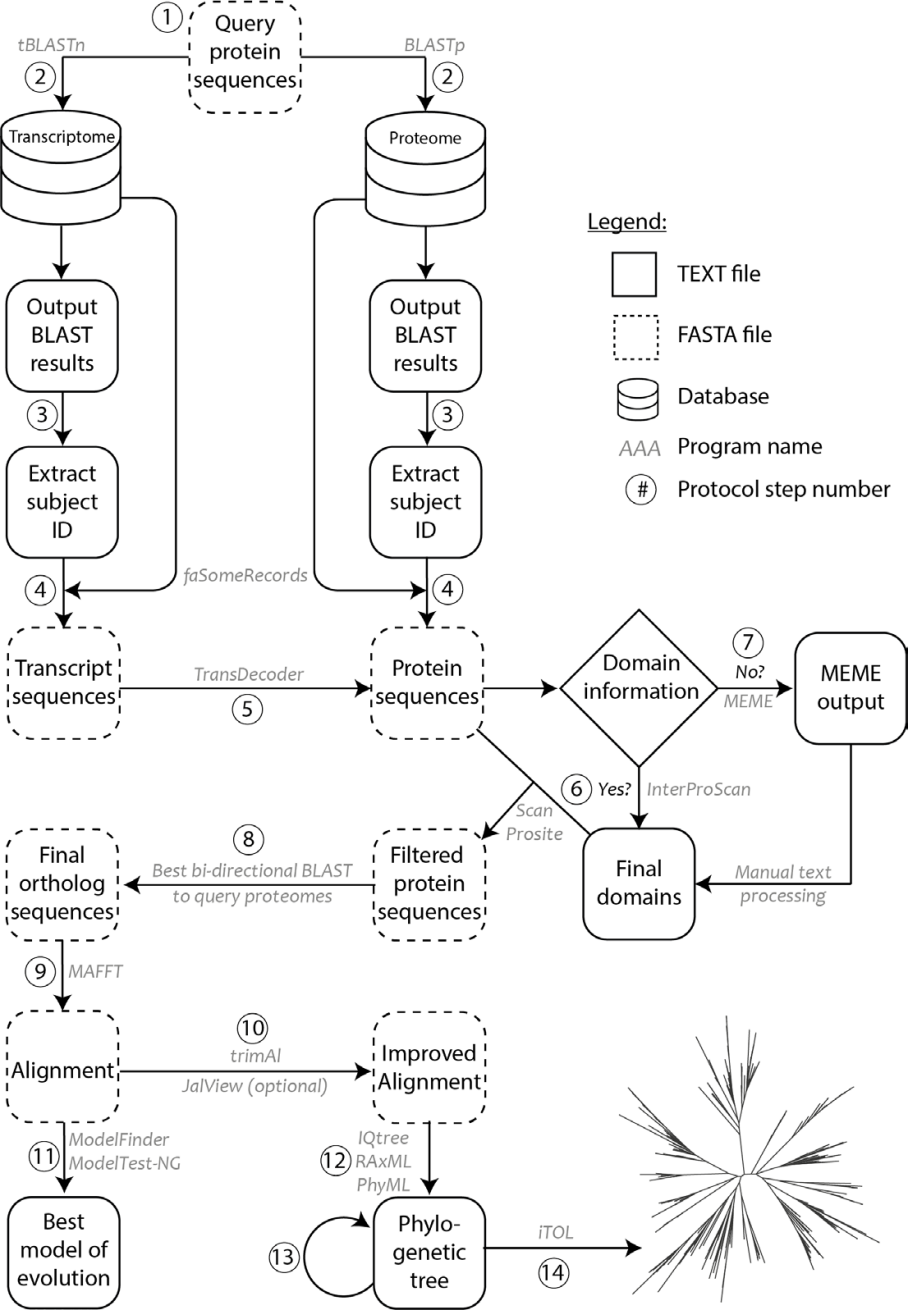
Methodology schematic showing various steps of the protocol used for ortholog identification and phylogenetic tree construction. Circled numbers correspond to the various steps of the protocol as indicated in the procedure. Programs/software/algorithms used are indicated next to the arrows in grey. File formats for text and FASTA are depicted as shown in legend.

Commands used, along with the parameters used in each step of the protocol, with step numbers corresponding to Figure 1 are given below. Before starting the protocol, we first created a BLAST database for each transcriptome or proteome. This was carried out only once for each transcriptome or proteome using the *makeblastdb* function, where ‘-in’ takes a FASTA file of the transcriptome, or the proteome and ‘-dbtype’ is the database type with nucl and prot for transcriptomes and proteomes, respectively.

$ makeblastdb -dbtype nucl -in transcriptome.fasta

$ makeblastdb -dbtype prot -in proteome.fasta

- Homolog identification
  1. To perform a BLAST search to the respective database(s), we created a query protein sequence file (in FASTA format), with sequences from (relatively) well-annotated genomes and from a diverse range of species, if present, across multiple kingdoms. A list of various species used along with a link to the sequence data resource is available in Appendix-1.
  2. Using the query sequence file (-query) perform the BLAST search with *tblastn* and *blastp* modules, against transcriptome and proteome databases (-db), respectively. When the E-value cut-off (-evalue) is less than 0.01, save the output (-out) in a tab-delimited text file indicated with -outfmt 6 . The remainder of the parameters are kept at default settings.
    $ tblastn -query filename.fa -db transcriptome.fasta -out output.blast -evalue 0.01 -outfmt '6 qseqid sseqid slen qstart qend sstart send evalue bitscore score length pident nident positive ppos mismatch gaps frames qcovs qcovhsp sseq'
    $ blastp -query filename.fa -db proteome.fasta -out output.blast -evalue 0.01 -outfmt '6 qseqid sseqid slen qstart qend sstart send evalue bitscore score length pident nident positive ppos mismatch gaps frames qcovs qcovhsp sseq'
  3. The BLAST output contains all the scoring information about the subject (transcript/protein) sequence that has a similarity to the corresponding query sequence. To retrieve the subject sequence identifiers from the BLAST output, we have used the *‘cut’, ‘sort’* and *‘uniq’* functions of a Linux BASH shell (terminal). *‘cut’* takes the BLAST output (output.blast) from the previous step, and takes the second column (-f2), *i.e.*, subject sequence identifiers and sends/pipes them (|) to the *‘sort’* function. After sorting, they are passed on to the *‘uniq’* function to remove the duplicates and the output is written to the file (SubjectIdentifiers.txt).
    $ cut -f2 output.blast | sort | uniq > SubjectIdentifiers.txt
  4. Using these identifiers (SubjectIdentifiers.txt) to extract the corresponding transcript (SelectedTranscripts.fasta) or protein sequences (SelectedProteins.fasta) from the respective transcriptome or proteome by running the ‘*faSomeRecords’* program.
    $ faSomeRecords transcriptome.fasta SubjectIdentifiers.txt SelectedTranscripts.fasta
    $ faSomeRecords proteome.fasta SubjectIdentifiers.txt SelectedProteins.fasta
  5. The protein sequences are more informative due to the higher number of site patterns and can be directly used for phylogeny construction. Whereas, the transcript sequences should be translated to protein sequences using the program *TransDecoder* with default settings. First, determine the longest Open Reading Frames (ORFs of at least 100 amino acids in length) of the transcript by TransDecoder.LongOrfs. And then the CDS and the corresponding amino acid sequences of these ORFs thorugh TransDecoder.Predict. If the tree based on protein sequences result in poor bootstraps, we would suggest generating the tree with CDS (DNA) sequences.
    $ perl TransDecoder.LongOrfs -t SelectedTranscripts.fasta
    $ perl TransDecoder.Predict -t SelectedTranscripts.fasta
- Ortholog detection
  1. Not all the sequences that have an E-value < 0.01 are true orthologs of a query protein. Hence, additional filters are needed to remove non-orthologs. One such filter is the presence of the same domains in orthologous proteins. For some well-annotated proteins (*e.g.*, Auxin Response Factors, Kinases, *etc.*), domain information is readily available in the *InterPro* domain database. Scan the protein sequences from the previous step (-i SelectedProteins.fasta) for the presence of known domains using *InterProScan* tool (interproscan.sh), which produces a tab-delimited (TSV) file as well as HTML/XML files (-f TSV,HTML,XML), with all the domains identified along with the corresponding InterPro identifiers (-iprlookup) in each protein sequence. A Python script was developed (InterproscanSummary.py) to process this TSV file, in order to extract the final set of protein sequences that have the domains of interest (See GITHUB page for more details). *InterProScan* is a time-consuming step, hence we used pre-annotated data where available, or reduced the number of databases to scan (using -appl Pfam,CDD setting), in order to save time. In some cases, we split the data in smaller batches and ran on multiple processors.
    $ interproscan.sh -f TSV,HTML,XML -iprlookup -i SelectedProteins.fasta
    $ python InterproscanSummary.py
  2. Certain proteins (*e.g.*, SOSEKI in *Arabidopsis*; Yoshida *et al.*, 2019) lack annotated (functional) domain information. Use the *MEME* program to predict the conserved motifs/domains in those proteins with Zero or One Occurrence Per Sequence criteria (-mod zoops) and a minimum width of 10 (-minw), with a maximum of 10 motifs predicted per set (-nmotifs). The *MEME* outputs the motifs along with their patterns in HTML/TEXT format. Then use these motif patterns in *ScanProsite* web-tool to identify the domains in the protein sequences that do not have annotated domains. We have applied this approach successfully to annotate the SOSEKI protein family and identify its orthologs (van Dop *et al.*, 2020).
    $ meme ProteinSequences.fa -o OutputName -protein -mod zoops -nmotifs 10 -minw 10
  3. After selecting the protein sequences that have the domains of interest, they are queried back to the proteomes of the species used in Step A1 to confirm the orthologous relationships using the best Bi-directional BLAST Hits (BBH) strategy. Here we have used the option of maximum target sequences or the number of best hits in the output (-max_target_seqs) set to 1, or sometimes 2 when domains are abundant in the genome (for *e.g.*, bHLH), with E-value < 0.01 (-evalue). This final set of proteins that have hits with the protein under consideration are regarded as the ‘true’ orthologous proteins for further analysis. Output is recorded in a TSV files, same as in Step A2 (-outfmt 6).
    $ blastp -query filename.fa -db ArabidopsisProteome.fasta -out BBhits.blastp -max_target_seqs 1 -evalue 0.01 -outfmt '6 qseqid sseqid slen qstart qend sstart send evalue bitscore score length pident nident positive ppos mismatch gaps frames qcovs qcovhsp sseq'
- Phylogeny construction
  1. These ‘true’ sets of orthologs are used for alignment followed by the phylogenetic tree construction. *MAFFT* is used to align protein sequences. The *E-INS-i (*--genafpair) algorithm is used while aligning proteins with multiple domains separated by poorly conserved sequences (*e.g.*, ARF or Aux/IAA proteins), whereas *G-INS-i (*--globalpair) is used while aligning only domain-specific sequences (*e.g.*, PB1 domain). An iterative refinement method is used in both cases, with a maximum of 1000 iterations (--maxiterate 1000), after which the final alignment is written to a FASTA file (output_file).
    $ mafft --genafpair --maxiterate 1000 input_file > output_file
    $ mafft --globalpair --maxiterate 1000 input_file > output_file
  2. Once the alignments are generated, use the *trimAl* to remove the sequence positions (columns) with more than 50%-80% gaps, as they are considered to lack phylogenetic signal. Hence, for phylogenetic tree construction, only use the sequences without spurious gaps. A gap-threshold of 0.2 (-gt 0.2), is set to remove all positions in the alignment with gaps in 80% (or more) of the sequences. For the gene families that have moderately conserved domains (*e.g.*, ARF, Aux/IAA), use a threshold of 0.3 or 0.4, whereas for poorly conserved domains (*e.g.*, PB1) it is set at 0.2, and for highly conserved proteins (*e.g.*, ROP, ROPGEF) it is set between 0.6 and 0.8. An additional (optional) check is kept in place, where the sequences that are shorter than 1/4^th^ of the average sequence length are further removed in *JalView*.
    Note: There are various tools specialized for the clean-up of the alignment, such as GBlocks, Guidance, AliScore, ZORRO etc. However, a simple gap-based trimming in trimAl resulted in (almost) the same quality of alignment and tree topology when compared to these specialized tools. Hence, we used trimAl for alignment clean-up throughout this study.
    $ trimal -in inputfile.fa -out outputfile.fa -fasta -gt 0.2
  3. Then use this ‘clean’ alignment to identify the most appropriate model of evolution for each protein family. *ModelFinder* and *ModelTest-NG* are used to predict the best model based on the Akaike- and Bayesian- Information Criterion (AIC and BIC). For the majority of the protein families, both programs provide the same models as the best models. The situations where there is a mis-match between the two programs, use a third program (either *PartitionFinder* or a Perl script from *RAxML* distribution) to decide on the best model based on the majority rule. As expected, various proteins evolve differently, leading to different models of evolution. *ModelFinder* is run as a part of IQ-TREE, hence it does not require any additional steps. *ModelTest-NG* requires the type (either amino acid or nucleotide -d) of input dataset (-i INFILE) and writes the statistics and the best model to the output file (-o OUTFILE). *PartitionFinder* requires the alignment, in the *PHYLIP* format (instead of *FASTA* format as in others) placed in the folder ‘partition_finder_models’, where the output statistics and best model are also recorded. *FASTA* to *PHYLIP* format conversion can be made through the Perl script (fasta2relaxedPhylip.pl), which takes input *FASTA* (-f input.fa) and writes the output in *PHYLIP* format (-o output.phylip).
    $ modeltest-ng -d aa -i INFILE -o OUTFILE
    $ perl RAxML_ProteinModelSelection.pl alignment.fasta
    $ perl fasta2relaxedPhylip.pl -f input.fa -o output.phylip
    $ python PartitionFinderProtein.py partition_finder_models
  4. Phylogenetic trees are built mainly using *IQ-TREE* and *RAxML* based on the ‘clean’ alignment produced in Step C10 and the evolutionary model predicted in Step C11. For the phylogenetic trees made through *IQ-TREE*, we have used 1,000 rapid bootstraps (-bb 1,000) and SH-like approximate Likelihood Ratio Test (-aLRT 1,000), combined with automatic model finding through *ModelFinder* (-m MFP+MERGE). For the trees made with RAxML, we have also used rapid bootstrapping and Maximum Likelihood search in the same run (-f a) but with an extended majority rule (-# autoMRE) based bootstopping criteria. In addition, we gave a random seed number (-x and -p) to turn-on rapid bootstrapping and parsimony inference, whereas -m takes in the model from the previous Step C11. For trees with very poor bootstrap support for majority of the branches, we used another phylogenetic tree construction program, *PhyML*, with 100 bootstrap replicates (-b 100), empirical amino-acid frequencies (-f e), gamma shape parameter estimated from maximum likelihood (-a e) and the topology was searched based on the sub-tree pruning and re-grafting approach (-s SPR). After running these multiple programs, the trees obtained were compared to understand the overall topology based on the congruent branches (see next step). We have also tried and tested various Bayesian approaches (using *MrBayes*), but the trees never converged even after months of computation, and provided various incongruent topologies. Hence, all the analyses were performed with Maximum Likelihood approaches.
    $ iqtree -s CleanAlignment.fa -pre OutputName -alrt 1000 -bb 1000 -m MFP+MERGE
    $ raxmlHPC-PTHREADS-AVX2 -f a -x 12345 -p 12345 -j -# autoMRE -m PROTGAMMAJTT -s CleanAlignment.fa -n OutputName
    $ PhyML-3.1_linux64 -i CleanAlignment.fa -d aa -b 100 -m JTT -f e -s SPR -a e
  5. Visualize all the final phylogenetic trees using the iTOL webserver and then various datasets on the phylogenetic trees. Generate protein domain information from the *InterProScan* or *MEME*, sequence length from *TransDecoder* and clade/taxonomy information from OneKP and MMETSP databases following the instructions provided in the iTOL documentation.
  6. Once the trees are obtained, they are manually checked for errors. Manually remove the branches with long branch attraction, or partial sequences or any misplaced taxa. If the proportion of these misplaced branches is too high, re-analyze the phylogeny with more sequences from other species, as well as by removing the spurious sequences. These steps are repeated until obtain better trees that are not only supported by good bootstraps but also obeys the taxonomy of those phyla.
  **Limitations and Conclusions**
  Due to the generalized nature of the method, it was difficult to automate the complete protocol. Hence, wherever possible, the method was simplified with scripts/commands dedicated for fast and parallel processing. On the other hand, it gave control over the decision-making process based on the protein under consideration. When dealing with highly redundant protein families, we removed highly similar proteins (> 90% similarity), prior to phylogeny, which reduced the (computational) time without losing accuracy. In many cases we observed that the best-hit in *reciprocal-BLAST* is not really a BBH, as sometimes a second hit was still the best one due to one or few amino acid difference(s) (especially in proteins with common domains *e.g.* bHLH or PB1). Hence, in those cases we considered two best hits and used both for phylogeny construction. The false positive orthologs were eventually placed in the outgroup (or at least separate from the ingroup) in the phylogenetic tree. As we were dealing with transcriptomes, we could not predict the actual gene copy number in each species, but only the ancestral copy number for that class or phylum, by comparing the ancestral copies across the majority of the species in that phylum. Another issue of dealing with (low-depth) transcriptomes was that we found many partial transcripts leading to the truncated proteins/domains, or we might fail to identify the transcripts that were not expressed in that particular tissue or condition. In that regard, combining ortholog sequence information from multiple transcriptomes or species of various families is mandatory to confirm the ancestral state for each class or phylum.
  Based on this protocol and the guidelines mentioned above, we have reconstructed the ancestral states of various protein families along with their orthologs in a ‘deep’ phylogenetic space, across multiple kingdoms. We demonstrated how this method was implemented for proteins that are well-defined with known domains, novel proteins with unknown domains, poorly conserved domains and phylum/kingdom-specific proteins that (dis)appeared at various stages in evolution. This approach was successfully applied for the core proteins of the auxin signalling (Nuclear Auxin Pathway (NAP)) and biosynthesis pathways. NAP includes Auxin Response Factor (ARF), Auxin/Indole-3-Acetic-Acid (Aux/IAA) and Transport Inhibitor Response 1/Auxin-signalling F-Box (TIR1/AFB; Mutte *et al.*, 2018). Biosynthesis pathway proteins include TAA family of amino transferase (TAA) and YUCCA family of monooxygenases (YUC). It was also applied to the individual domains, Phox and Bem1 (PB1; Mutte and Weijers *et al.*, 2020), along with various downstream targets of the auxin pathway, such as SOSEKI (SOK; van Dop *et al.*, 2020), Target of MOnopteros 5 (TMO5) and its interaction partner Lonesome HighWay (LHW; Lu *et al.*, 2020). Taken together, by following this protocol in combination with ever-growing high-quality sequence data, and leaping developments in the methods and algorithms in phylogenetics, reveal new evolutionary insights into our understanding of proteins and the crucial pathways.
