# Supplementary material for: High-resolution and Deep Phylogenetic Reconstruction of Ancestral States from Large Transcriptomic Data Sets
Source: Bio Protoc. 2020 Mar 20;10(6):e3566. doi: 10.21769/BioProtoc.3566 (PMC7842344; doi:10.21769/BioProtoc.3566)
Supplement: Appendix-1 [file BioProtoc-10-06-3566-s001.docx]

**Appendix-1:** Recommended species to get query sequences from well annotated genomes

| Plant | *Arabidopsis thaliana* | <https://phytozome.jgi.doe.gov/pz/portal.html> |
| --- | --- | --- |
|  | *Oryza sativa* | <https://phytozome.jgi.doe.gov/pz/portal.html> |
|  | *Amborella trichopoda* | <https://phytozome.jgi.doe.gov/pz/portal.html> |
|  | *Picea abies* | <http://congenie.org/> |
|  | *Physcomitrella patens* | <https://phytozome.jgi.doe.gov/pz/portal.html> |
|  | *Marchantia polymorpha* | <https://phytozome.jgi.doe.gov/pz/portal.html> |
|  | *Chara braunii* | <https://bioinformatics.psb.ugent.be/orcae/overview/Chbra> |
|  | *Klebsormidium nitens* | <http://www.plantmorphogenesis.bio.titech.ac.jp/~algae_genome_project/klebsormidium/> |
| Animals | *Homo sapiens* | UniProt: UP000005640 |
|  | *Mus musculus* | UniProt: UP000000589 |
|  | *Gallus gallus* | UniProt: UP000000539 |
|  | *Strongylocentrotus purpuratus* | UniProt: UP000007110 |
|  | *Caenorhabditis elegans* | UniProt: UP000001940 |
|  | *Drosophila melanogaster* | UniProt: UP000000803 |
| Fungi | *Aspergillus nidulans* | <https://mycocosm.jgi.doe.gov/mycocosm/home> |
|  | *Schizosaccharomyces pombe* |  |
|  | *Saccharomyces cerevisiae* |  |
|  | *Agaricus bisporus* |  |
|  | *Mortierella elongate* |  |
|  | *Rhizoclosmatium globosum* |  |
| Protozoa | *Dictyostelium discoideum* | UniProt: UP000002195 |
|  | *Entamoeba histolytica* | UniProt: UP000001926 |
|  | *Leishmania major* | UniProt: UP000000542 |
|  | *Monosiga brevicollis* | UniProt: UP000001357 |
|  | *Trypanosoma brucei* | UniProt: UP000008524 |
